# Supplementary material for: A set of multi-entry identification keys to African frugivorous flies (Diptera, Tephritidae)
Source: Zookeys. 2014 Jul 24;(428):97–108. doi: 10.3897/zookeys.428.7366 (PMC4143993; doi:10.3897/zookeys.428.7366)
Supplement: Supplementary material 6 — Key to Ceratitis [file zookeys-428-097-s006.zip › SF6_ZooKeys_key to Ceratitis/key/SF6_ZooKeys_key to Ceratitis/Media/Html/desc_fake.html]

Natural Language Description


# A multi-entry identification key to African frugivorous fruit flies (Diptera, Tephritidae)

### Massimiliano Virgilio, Ian White, Marc De Meyer

## fake

(key to Ceratitis) sex male or female. (key to Ceratitis) head (key to Ceratitis) 2. first flagellomere yellow to red or brown to black, (key to Ceratitis) 3. arista hairing bare, short hairs, medium long hairs or distinct plumose, (key to Ceratitis) 4. (males) frons enlarged and inflated yes or no, normal in shape, (key to Ceratitis) 5. frons longer than wide no (as wide or wider than long) or yes, (key to Ceratitis) 6. frons with silvery shine, at least in some areas yes or no, (key to Ceratitis) 7. (males) ocellar tubercle on protuberance yes or no, (key to Ceratitis) 8. ocellar setae colour black or pale, (key to Ceratitis) 9. frontal setae yes or no, (key to Ceratitis) 10. number of frontal setae one, two or three, (key to Ceratitis) 11. upper frontal setae well developed yes or no (poorly developed), (key to Ceratitis) 13. (males) superior orbital setae incrassate yes or no, normal, (key to Ceratitis) 14. (males) lower orbital setae incrassate, flattened to some degree yes or no, (key to Ceratitis) 15. (males) modified lower orbital setae distinctly spatulate, apex at least twice as broad as stem yes or no, (key to Ceratitis) 16. (males) spatulate apex at least 3 times longer than broad yes or no, (key to Ceratitis) 17. (males) spatulate apex pale or dark, (key to Ceratitis) 18. (males) modified lower orbital setae length about as long as arista, at most one-third length arista or at least twice length arista, (key to Ceratitis) 19. face, ground colour white to yellow or orange to red, (key to Ceratitis) 20. face with transverse median band yes or no, (key to Ceratitis) 21. face with upper half darker coloured yes or no, (key to Ceratitis) 22. lower margin of face same colour as rest of face yes or no (at least looked at from certain angle), (key to Ceratitis) 23. lateral vertical setae black yes or no, pale, (key to Ceratitis) 24. mouthparts partly or wholly brown to black or no, pale, at most isolated dark patches in upper part, (key to Ceratitis) 25. occiput in upper part dark yes or no, (key to Ceratitis) 26. head with 2 orbital setae yes or no, 1 or 3, (key to Ceratitis) 27. frons with medilongitudinal shining line yes or no. (key to Ceratitis) thorax (key to Ceratitis) 28. postpronotal lobe with distinct brown or black spot yes or no, at most darker yellow, (key to Ceratitis) 29. spot occupying most of postpronotum yes or no, smaller, (key to Ceratitis) 30. scutum dark streaks or spots clearly defined dark streaks or spots, unclear streaks and darker markings; markings usually obvious when viewed from behind, obscured when viewed from front or no spots or streaks, (key to Ceratitis) 31. scutum with a dark lateral stripe that includes both the anterior supra-alar seta and the intra-alar seta yes or no, (key to Ceratitis) 32. (male) scutum with anterior third darker, sickle-shaped dark coloration yes or no, (key to Ceratitis) 33. postsutural (pale or yellow) lateral lines present yes or no, (key to Ceratitis) 34. prescutellar markings present yes or no, (key to Ceratitis) 36. scutellum colour form unicolorous or marked, (key to Ceratitis) 37. scutellum markings trilobed (joint or separate), five separate dots or largely black with subbasal pale spots, (key to Ceratitis) 38. scutellum with five separate dots of equal size yes or no, three large apical spots and two central spots, (key to Ceratitis) 39. base scutellum with 2 brown or black spots yes or no, at most localized darker coloring, (key to Ceratitis) 40. spots at base scutellum joining yes or no, (key to Ceratitis) 41. spots at end scutellum separate, joining or separate, except at apical margin joining, (key to Ceratitis) 42. joint spots at end scutellum incised deeply incised, almost reaching apical bristles, only weakly incised or incised till halfway, (key to Ceratitis) 43. apical spots extent reaching till basal third or fourth of scutellum, only reaching till halfway of scutellum at most or restricted to apical margin only, (key to Ceratitis) 44. anepisternal setae one, two or three, (key to Ceratitis) 45. anepisternum pilosity whole pale pilosity, except few darkish hairs along upper margin, partly dark pilosity in lower half or mainly dark pilosity over entire length, or almost so, (key to Ceratitis) 46. anepisternum colour completely pale, lower half darker coloured or lower margin darker coloured, (key to Ceratitis) 47. lower part anepisternum brown to black or darker yellow to orange, (key to Ceratitis) 48. large black prescutellar-acrostichal spots yes or no, absent, (key to Ceratitis) 49. prescutellar-acrostichal and dorsocentral black spots merged to one large spot yes or no, (key to Ceratitis) 50. merged prescutellar-acrostichal-dorsocentral black spot linked with posterior lateral black line through small bridge yes or no, both black areas separate, (key to Ceratitis) 51. median sutural spot present yes, sometimes as continuous longitudinal line or no, (key to Ceratitis) 52. median postscapular spot present yes, sometimes as continuous longitudinal line or no. (key to Ceratitis) wings (key to Ceratitis) 53. wing with anterior apical band forming continuous band with anterior part of discal band (at most connection partly interrupted) yes or no, (key to Ceratitis) 54. discal band width narrower, not joined with basal spots or very broad, joined with basal spots, (key to Ceratitis) 55. subapical band free, joined with anterior apical band or joined with discal band, (key to Ceratitis) 56. posterior apical band joined with anterior apical band, free or absent, or poorly developed, (key to Ceratitis) 57. wing with longitudinal band along vein M yes or no, (key to Ceratitis) 58. wing banding reduced to isolated spot(s) yes or no, (key to Ceratitis) 59. crossvein r-m placed at middle of cell dm, before middle of cell dm or beyond middle of cell dm, (key to Ceratitis) 60. (males) wing with subapical incision lobe yes or no, (key to Ceratitis) 61. (males) wing apical tip shape pointed or rounded, (key to Ceratitis) 62. wing apical tip with isolated spot yes or no. (key to Ceratitis) legs (key to Ceratitis) 63. fore coxa black yes or no, (key to Ceratitis) 64. fore coxa with silvery patch yes or no, (key to Ceratitis) 66. (males) fore femur with anteroventral dense row of silvery hairs yes or no, (key to Ceratitis) 67. (males) fore femur posteriorly with bush of hairs yes or no, (key to Ceratitis) 68. (males) fore femur posterior bush colour black, orange or pale, (key to Ceratitis) 69. (males) mid femur, ventrally with feathering (even only partly) yes or no, (key to Ceratitis) 70. (males) gap in feathering large yes or no, (key to Ceratitis) 71. (males) mid femur, ventral feathering with gap yes or no, (key to Ceratitis) 73. (males) mid tibia with feathering yes or no, (key to Ceratitis) 74. (males) mid tibia feathering extension in apical part reaching less than half-way, reaching half way but less than 3/4 or reaching beyond 3/4, (key to Ceratitis) 75. (males) feathering on mid tibia - coloured dark or orange, (key to Ceratitis) 76. (males) mid tibia flattened and broadened yes or no, (key to Ceratitis) 77. (males) mid tibia anterior side with long (and dispersed) hairs yes or no, (key to Ceratitis) 77bis. (males) mid tibia anterior side with long (and dispersed) hairs yes or no, (key to Ceratitis) 78. (males) mid tibia anterior side with silvery shine when viewed from certain angle yes or no, (key to Ceratitis) 79. (females) fore femur with dark hairs between posterior and posterodorsal row of bristles yes or no. (key to Ceratitis) abdomen (key to Ceratitis) 80. abdomen spot pattern spotted, unspotted or spotted but spots unclear, (key to Ceratitis) 81. tergite 2 with silvery grey band yes or no, (key to Ceratitis) 82. tergite 4 with silvery grey band yes or no, (key to Ceratitis) 83. tergite 3 with brown-black band or completely black yes or no, (key to Ceratitis) 84. (females) oviscape length shorter than abdominal tergites, at most as long, at least as long as abdominal tergites, usually longer or very long, at least twice as long as abdominal tergites, (key to Ceratitis) 85. (females) aculeus tip simple, without notch, identations, serrations or appendages yes or no, (key to Ceratitis) 86. (females) aculeus tip with small notch only and no indentations, serrations or appendages yes or no, modified in different way, (key to Ceratitis) 87. (females) aculeus pointed, tip with subapical step only and no indentations, serrations or appendages yes or no, (key to Ceratitis) 88. (females) aculeus tip uniformly serrated yes or no, modified in different way, (key to Ceratitis) 89. (females) aculeus tip serrated and with lateral protuberances above the serrations yes or no, modified in different way, (key to Ceratitis) 90. (females) aculeus tip serrated with large blad-like serrations yes or no, (key to Ceratitis) 91. (females) aculeus tip with large indentations, broadening upwards yes or modified in different way, (key to Ceratitis) 92. (females) aculeus distal end elongated but straight yes or no, not as such.
